# Autonomous Aeromagnetic Surveys Using a Fluxgate Magnetometer

**DOI:** 10.3390/s16122169

**Published:** 2016-12-17

**Authors:** Douglas G. Macharet, Héctor I. A. Perez-Imaz, Paulo A. F. Rezeck, Guilherme A. Potje, Luiz C. C. Benyosef, André Wiermann, Gustavo M. Freitas, Luis G. U. Garcia, Mario F. M. Campos

**Affiliations:** 1 Department of Computer Science, Universidade Federal de Minas Gerais, Belo Horizonte 31270-901, Brazil; hector.azpurua@dcc.ufmg.br (H.I.A.P.-I.); rezeck@dcc.ufmg.br (P.A.F.R.); guipotje@dcc.ufmg.br (G.A.P.); mario@dcc.ufmg.br (M.F.M.C.); 2 Observatório Nacional/MCTI, Coordenação de Geofísica, Rio de Janeiro 20921-400, Brazil; benyosef@on.br (L.C.C.B.); andrew@on.br (A.W.); 3 Vale Institute of Technology, Ouro Preto 35400-000, Brazil; gustavo.medeiros.freitas@itv.org (G.M.F.); luis.uzeda@itv.org (L.G.U.G.)

**Keywords:** geophysical surveys, fluxgate magnetometer, autonomous vehicles, rotary-wing UAVs, drones in mining applications

## Abstract

Recent advances in the research of autonomous vehicles have showed a vast range of applications, such as exploration, surveillance and environmental monitoring. Considering the mining industry, it is possible to use such vehicles in the prospection of minerals of commercial interest beneath the ground. However, tasks such as geophysical surveys are highly dependent on specific sensors, which mostly are not designed to be used in these new range of autonomous vehicles. In this work, we propose a novel magnetic survey pipeline that aims to increase versatility, speed and robustness by using autonomous rotary-wing Unmanned Aerial Vehicles (UAVs). We also discuss the development of a state-of-the-art three-axis fluxgate, where our goal in this work was to refine and adjust the sensor topology and coupled electronics specifically for this type of vehicle and application. The sensor was built with two ring-cores using a specially developed stress-annealed CoFeSiB amorphous ribbon, in order to get sufficient resolution to detect concentrations of small ferrous minerals. Finally, we report on the results of experiments performed with a real UAV in an outdoor environment, showing the efficacy of the methodology in detecting an artificial ferrous anomaly.

## 1. Introduction

Robotics has received notoriety in our society mainly due to the wide use of manipulators. However, recent advances on the research of autonomous vehicles have shown a much more vast range of applications, such as exploration, surveillance and environmental monitoring. In this sense, the use of aerial robots have a clear advantage over other kind of robots, since they are able to navigate over large areas faster and with a privileged view from above.

Autonomous mapping in Robotics could be defined as the task of acquiring models of the environment using autonomous mobile robots [1]. One of the most versatile type of mapping is the aerial one. Aerial surveys can use a great variety of sensors such as cameras, lasers, radio receptors, barometers, magnetometers, etc. Currently, most aerial surveys are created using a manned aircraft with expensive and special equipment, and therefore it is a high cost and time consuming task, as it generally must be done on large areas. In this context, autonomous aerial robots can bring many benefits to the workers and companies. Mapping with autonomous robots can reduce the risk of endangering human lives, and it can also reduce the economic costs of the task.

In this context, geophysical maps such as magnetic, gravitational and textured digital elevation maps can be used in different situations. Some practical applications that benefit from such maps are: (i) in geology it is possible to use a coverage strategy to detect minerals of commercial interest beneath the ground; (ii) in the military, to find unexploded ordnance (UXOs), sunken ships and submarines at sea; (iii) in archeology, they are used to create maps of subsurface buried artifacts; among others.

One of the main aspects that must be considered before executing a mapping is which type of information must be acquired, and consequently the sensors that will be used. In geophysical surveys, magnetometers are among the most widely used ones, allowing for the evaluation of the magnetic field at a certain region.

The fluxgate magnetometer is a traditional and well known instrument that have been used in geophysical exploration for decades, mostly due to its small size, good sensitivity, low noise and relatively low power consumption. This low noise characteristic is however directly related to a strong limit on its frequency response.

Fluxgate sensors are built using materials of high magnetic permeability that are excited up to the saturation limits by a source with periodic current. A significant part of this noise is due to magnetic properties of the sensor core. The right choice of materials and special thermal treatment can produce sensors with very low noise figures. The characteristics of the core material are also fundamental to improve the intensity of the induced signal in the sense coil [2]. There are several new amorphous and crystaline (nanocrystaline) materials available [3,4,5], and deeper investigation would be necessary to see if this class of material has the potential to be used in fluxgate applications.

In most uses as in geomagnetic stations, the fluxgate integration filter is set to frequencies usually below 1 Hz, with 0.1 Hz being typical [6], which is not adequate for moving platform applications. To increase the sensor frequency response, a higher frequency is also necessary for the core excitation, consequently affecting the core material choice. Cobalt based amorphous alloys are among the best for fluxgate sensor due to its lower intrinsic noise and lower conductivity which result in smaller eddy currents, favoring its use at higher frequencies [7].

In this work, we discuss the development of a state-of-the-art three-axis fluxgate magnetometer to be used in aeromagnetic surveys. The proposed sensor is specially designed for small rotary-wing UAVs, considering possible applications in the mining industry such as prospection of ferromagnetic minerals beneath the ground.

The magnetometer uses two ring-cores with CoFeSiB amorphous ribbon developed for the detection of small ferrous minerals concentration. Due to UAV restricted resources, the electronic design aims to reduce its size and power consumption. The sensor housing is assembled far from the electronics, in order to allow enough distance from the UAV’s motors, a substantial source of magnetic noise.

The remainder of this paper is structured as follows: Section 2 presents the details regarding the magnetometer development. The coverage path planning strategy for autonomous mapping is proposed in Section 3, followed by Section 4 in which we describe the methodology for data acquisition and map generation. The experiments and analysis about the results are presented in Section 5 and finally in Section 6 we draw the conclusions and discuss paths for future investigation.

## 2. Magnetometer Development

Detailed geophysical surveys need accurate sensor information. These mapping executions with aerial techniques are very restricted. In most cases, such missions are done manually using a manned aircraft, which increases the survey duration, as well as the economical costs, whilst the quality of data is not necessarily improved. Other types of magnetic surveys can be done by foot or by ground vehicles, thus augmenting the survey time.

On the other hand, mapping with autonomous robots can reduce risks and costs related to the task. Due to the UAV’s potential to increase productivity while mitigating safety hazards, mining companies already benefit from such equipment integrated into their operations.

The sensor described here is designed specifically for mining applications, focusing on aeromagnetic surveys for prospection of ferromagnetic minerals beneath the ground. The instrument is capable to detect small ferrous minerals concentration, and suitable for installation in moving platforms. Considering the UAV restrictions, the developed magnetometer presents particular characteristics, including reduced weight, dimensions, and power consumption. We also decrease costs greatly by considering the use of small and inexpensive robots. The proposed sensor is at par with other fluxgate magnetometers on the market with a similar scale range and sensitivity [8,9] but with an increased data rate of ≈4–5 Hz and smaller size.

Table 1 presents a summary of the three-axis fluxgate magnetometer proposed to be used in autonomous aeromagnetic surveys.

**Figure 1:**
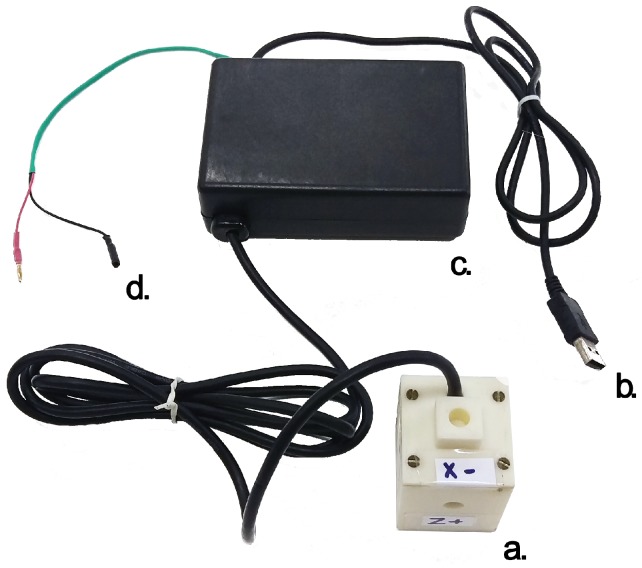
The developed sensor exposing the connectors. (**a**) The sensor core; (**b**) serial connector to acquire the data; (**c**) the Printed Circuit Board (PCB) protected by a small plastic case; and (**d**) power connectors.

Figure 1 shows the complete sensor mounting.

### 2.1. Materials

In this work, we used a ${\mathrm{Co}}_{67.5}{\mathrm{Fe}}_{3.5}{\mathrm{Si}}_{17.4}B_{11.6}$ composition melted by melt-spinner method [10] and after submitted to a convenient stress-annealing technique [11] increasing its sensibility and reducing its noise level.

As shown in the literature, there is a dependence between the magnetization process of the amorphous ribbon and the induced effects when an external load (“stress”) is applied on the ribbon during the adjustment process in the core [12]. So to reduce them is convenient to use the ring-core geometry as applied in this experiment.

The ribbon was improved using an adequate stress-annealing that is fundamental to induce the appearance of transverse magnetic anisotropy causing rotation of the spontaneous domains of magnetization. A domain study of magnetization processes in a stress-annealed metallic glass ribbon for fluxgate sensors [12] confirms the importance of the longitudinal axis of the amorphous ribbon to be an easy magnetization axis, as well as the need of a convenient rotation of the domain walls, to obtain low levels of noises in fluxgate sensors. When the ribbon is accurately thermally annealed, it makes possible to obtain a fluxgate sensor with low level of noise, with a performance superior to the commercial crystalline materials [13].

### 2.2. Instrument Construction

**Figure 2:**
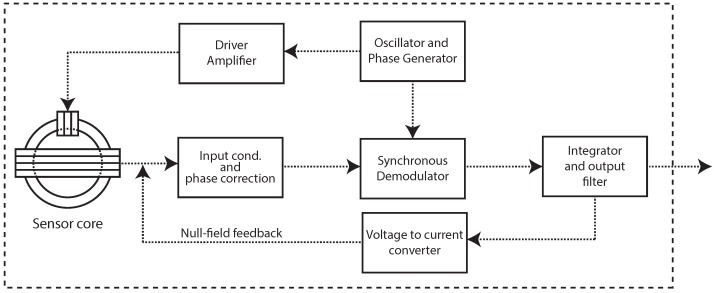
Block diagram of the sensor conditioning electronic circuit.

The electronic circuit devised for this application is based on the traditional topology of second harmonic synchronous demodulation with current feedback, with the sensor operating in a null field condition [14]. This arrangement allows for a large dynamic range and great stability, reducing the circuit sensitivity to core parameter variations. A simplified block diagram for the sensor processing circuitry is shown on Figure 2.

Although based on a traditional configuration, some minor changes were made to assure a good performance under fast changing field conditions as faced on a moving platform. No resonant filter was used at the sensor secondary winding and a special capacitive sampling and hold acted as a synchronous demodulator. All control pulses were produced by a small micro controller embedded in the oscillator and phase generation stage, conferring also great flexibility during the adjustment and calibration tasks. In order to reduce overall sensor size, two axes (X,Y) were wounded over one single core. This arrangement additionally saves 30% of drive power when compared to traditional three ring cores topology.

**Figure 3:**
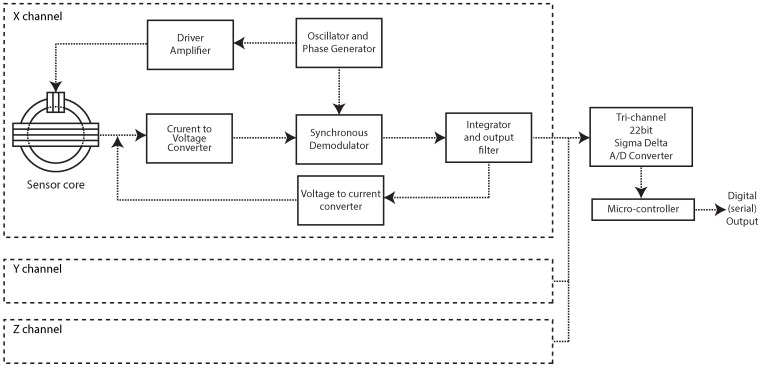
Complete magnetometer block diagram showing three channels and digital control.

After demodulation and integration, the analog signal from each axis is carried to a three channel 22 bit sigma-delta A/D converter, controlled by a small micro-controller responsible for taking the measurements, apply calibration coefficients and transmit the resultant information throughout a serial communication port as in Figure 3.

## 3. Autonomous Mapping

### 3.1. Related Work

Path planning is a fundamental task for any kind of autonomous mobile robot. This problem in the context of area coverage is a popular research topic in recent years due to its varied uses. It can be assumed that the quality of the mapping task (e.g., resolution, energy consumption, execution time, etc.) is very dependent of an efficient coverage, so defining efficient routes is mandatory in many circumstances.

To perform a correct coverage of a region with a robot, some requirements need to be fulfilled: the robot needs to be able to reach all the points conforming the area, traversing the region using simple and/or optimal paths without overlapping or repeating them, and avoiding any obstacles for a safe navigation [15]. In addition, there are other constraints related to the particular characteristics of the robots and the environment such as autonomy optimization and availability of prior knowledge about the region. Execution time and vehicle autonomy can be critical in defining what type of trajectory will be generated, as small unmanned robots may have very limited autonomy.

In the literature, several coverage patterns have been studied, and in between the most efficient ones there is the lawnmower pattern [16,17,18]. This pattern is simple to implement as it is composed by several parallel straight lines with a space between them.

Other works adapt ground coverage solutions for aerial coverage. In [19] the authors proposed the use Boustrophedon cell decomposition [20] on the region of interest to then generate a Euler circuit with all the connected cells. Finally a lawnmower path is used to comb the cells. A photometric mapping system with quad rotors was developed in [21]. The authors proposed a wavefront planner that uses square cell decomposition with a simple breadth-first search (BFS) on the resulting graph to generate the routes.

A very important aspect when dealing with large areas to cover and small robots is cooperation. Robotic cooperation can be defined as a group of robots working together to perform one or several tasks in order to increase the efficiency and robustness of the task.

**Figure 4:**
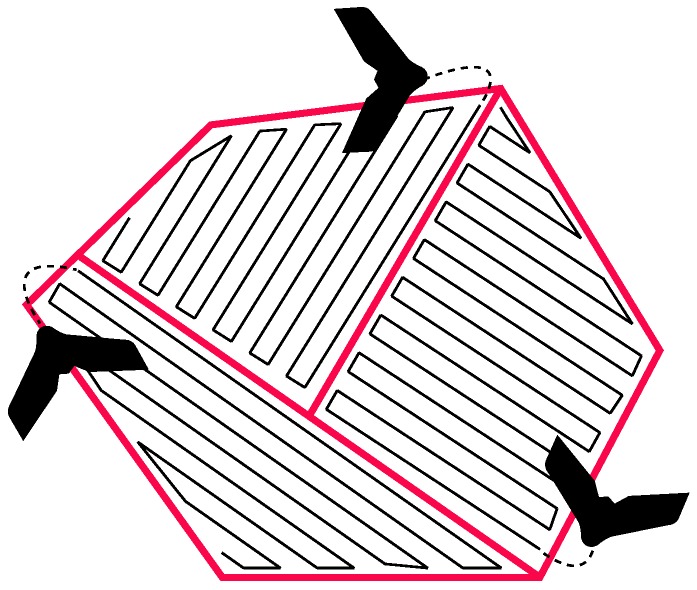
Example illustrating the method proposed in [22]. The area is decomposed using the robots autonomy and inside every cell the coverage pattern is optimized to decrease unnecessary turns.

Aerial cooperative coverage has been a great research area over the last years due the advances and miniaturization of components and sensors. In this context, the work of [22] is one of the first to propose a cooperative coverage approach using such vehicles. It uses a polygonal area decomposition with heuristics to assign the routes between the robots considering their autonomy limitations as shown in Figure 4. Next, it is generated a collection of straight lines with the maximum length possible with the assumption that robots do not waste time on curves and stay most of the time at full speed.

In [23] the authors developed a path planning algorithm for multiple Unmanned Aerial Vehicles (UAVs) also based on cell decomposition. The allocation of the cells depends on the sensor footprint of every aircraft. In [24] the region of interest is subdivided with an greedy algorithm which minimizes the number of turns inside every sub-area. The authors also proposed the generation of sweep lines with the biggest length possible, and then the visit order of the cells is calculated by using an undirected graph.

In [25] it is proposed an algorithm which does not consider the environment segmentation. Initially, a collection of waypoints is assigned to the boundary of the region of interest, next it is created a graph composed by these waypoints, finally the original problem is formulated as the Vehicle Routing Problem (VRP) [26]. The main contribution is the incorporation of specific features that are relevant in a real-world deployment, such as a setup time. However, they do not consider cases where the number of vehicles or their autonomy are not sufficient given the size of the area to be covered.

In this sense, this work proposes a novel magnetic survey pipeline, that aims to increase versatility, speed and robustness of surveys by autonomous mapping using UAVs. The proposed approach is angle configurable to match the magnetic north, and allows configure several other parameters to match different mapping conditions. Quality of the magnetic data is increased due the stationary acquisition on every survey point.

### 3.2. Methodology

For the particular task of finding and recognizing magnetic anomalies we propose the use of modified version of the Maza et al. [22] path planning algorithm. The path planning algorithm is based on the lawnmower coverage pattern, that is one of the most efficient ways to cover a region [16,17,18]. The coverage process is divided in 3 steps once a region of interest is defined: (i) Parameter definition; (ii) Path generation and optimization; and (iii) Feasibility verification. A region of interest is defined as area bordered by the vertexes list V = v1, v2, v3, ..., vn forming a polygon *P*, which we assume is convex.

#### 3.2.1. Parameter Definition

To allow the aircraft be able to fulfill the tight constrains of a conventional magnetic survey some parameters had to be defined before generating the coverage path.

- Coverage angle, since it is recommended to keep the sensor aligned with the magnetic north of the region, this parameter allows the aircraft to define a coverage angle and/or specific yaw when acquiring the magnetic data on hover.
- Separation between survey waypoints, depending of the survey resolution needed the distance between survey waypoints need to be greater or smaller. For small anomalies, this distance can vary from 1 m to 3 m. For greater anomalies the distance could be several hundred meters [27].
- Flight height, the quality of the magnetic sensing decay to the cube of the distance from the anomaly [27], so this parameter could make the entire survey campaign useless is settled very high. For a balance between great resolution and safety we fly at approx 15 m off the ground.
- Waypoint hover time, since the fluxgate magnetometers have a low acquisition rates, the robot will need to hover every waypoint for a specified time to be able to acquire information with less noise possible. We empirically choose to hover 5 s at every survey point due that it was enough time for the UAV to stabilize.

#### 3.2.2. Path Generation and Optimization

Once a region of interest and a set of parameters is defined, the survey path can be generated. The developed fluxgate sensor require the minimum movement possible to acquire low noise measurements. Quadrotors, unlike other aircraft such as fixed wing airplanes, can fly or “hover” over a specific point indefinitely. The survey path proposed subdivides the total survey path in small sections (generally 1 to 5 m) to allow the aircraft hover over every acquisition point, pointing the magnetic north an specified amount of time to collect enough readings to recreate that point the more accurately possible.
**Algorithm 1** Survey path (P,α,d)1:P←rotateCW(P,α)2:$\left(h_{min},h_{max},v_{min},v_{max}\right)\leftarrow getBoundaries\left(P\right)$3:$L\leftarrow getVerticalLines(v_{min},v_{max},d)$4:R←5:i=06:**while**
i<|L|
**do**7: $p=L_{i}$8: **if**
i+1<|L|
**then**9:  $p\leftarrow p\cup L_{i+1}$10:  i+=211: **else**12:  i+=113: **for**
j∈{0,...,|p|}
**do**14:  $K\leftarrow intersection(h_{min},h_{max},p_{j})$15:  D←subdivide(k,d)16:  **if**
(i+j)%2==0
**then**17:   D←reverse(D)18:  R←R∪D19: R←rotateCCW(R,α)20:**return**
*R*

**Figure 5:**
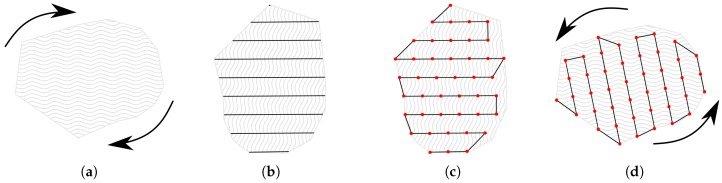
Survey path. (**a**) A region of interest is defined *P* and then rotated to be parallel to the *X* axis; (**b**) *P* is segmented in sections with an *d* distance; (**c**) Every line segment is subdivided in smaller *d* size segments and the visiting sequence is defined. Every survey visit point is denoted in red; (**d**) The final path is then rotated back to the desired angle.

The algorithm for survey path generation can be seen in Algorithm 1 and a result of the generated path can be seen in Figure 5. The input of Algorithm 1 is the *P* polygon denoting the region of interest, *α* coverage angle and *ω* width separation between survey points. The algorithm first rotate *P* to be aligned horizontally, and then it calculates the position of the survey lines. Then it defines the size of the survey line and it subdivides it in small *ω* segments. Finally it again rotates back to the desired *α* angle and return the *R* path with the survey points.

In special cases where the survey require to have a specially low distance between survey points, a particular localization problem arises with small aerial robots. The GPS sensor on many of those small and expendable robots have an accuracy of 1 to 2.5 m [28], therefore setting such small distance between points will make the robot to skip the points inside the error radius of their on-board GPS sensor.

**Figure 6:**
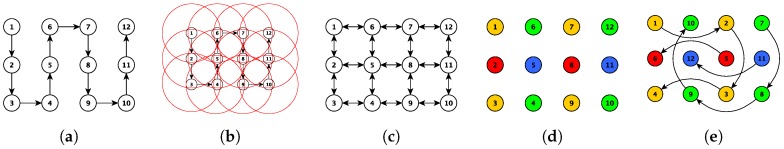
Spaced visiting sequence; (**a**) Normal survey path *R*; (**b**) A new graph is created, the red circles are the distance threshold to connect to neighbors; (**c**) Neighbors connected *G*; (**d**) Edges labeled by color; (**e**) Final path.

To tackle this problem we propose an algorithm that reorganize the visiting points allowing the aircraft to visit them with a minimum distance threshold to avoid those common GPS problems. The method can be observed in Algorithm 2 (Figure 6), it receives as input the *R* survey points and a *β* distance treshold. The algorithm first generate a graph where the vertexes are the survey points and the edges are created only for survey points that are inside a distance threshold. After that we used the DSATUR vertex coloring algorithm [29] to label the neighbor vertexes. The new generated group of labeled vertexes will not share any direct neighbors inside the distance threshold. Every set of vertexes is then optimized using a TSP (Traveling Salesman Problem) solver to obtain the visiting order that minimizes distance. Finally all the paths are concatenated: if there is any points closer than the distance threshold the process is repeated, else the new survey point sequence is returned.
**Algorithm 2** Spaced survey points (R,β)1:G←getGraphEdges(R,β)2:S←graphColoring(G)3:R←{}4:f←False5:**while**
*f* is *False*
**do**6: **for**
i∈{1,...,|S|}
**do**7:  $s\leftarrow S_{i}$8:  s←TSPSolver(s)9:  R←R∪s10: f←checkThreshold(R)11:**return**
*R*

#### 3.2.3. Feasibility Verification

Finally, in order to verify if the route can be completed by the robot, a time estimation is generated using the following equation:

$$t_{total}=\left(|R\right|\times t_{h})+(|R|\times d)/v,$$

where $t_{h}$ is the hovering time, *d* the distance between survey points and *v* the robot speed (m/s). Therefore, if $t_{total}$ is greater than the actual flight time of the robot, the path cannot be done with the specified robot.

## 4. Magnetic Map Generation

### 4.1. Data Acquisition Strategy

Once a path is generated by the algorithm described in Section 3, the aerial vehicle will obtain the mean of 10 reading samples from the magnetometer for each point in an approximate regular grid within the desired area of coverage. The grid is said to be approximately regular mainly because of the GPS errors (less than 2 m) and the vehicle position instability.

The GPS navigation proven to be superior in time and with a relative low error in comparison with other techniques such as markers. GPS navigation allow the robot to be deployed fast and reach regions where terrestrial locomotion is difficult or dangerous. GPS error position can be also improved to less than 5 cm error with the use of differential GPS systems.

Generally, the final results of a interpolation depend on the spatial distribution of the points to be interpolated. A regular grid profile [30] is a good choice for this scenario in order to avoid artifacts and distortions on the final map. An irregular profile caused by disproportional spacing between points, will not accurately represent the actual signal that originated the data. The sampling value was empirically selected to increase reading accuracy, and decrease the effects of the UAV displacement over the plane and vibrations.

**Figure 7:**
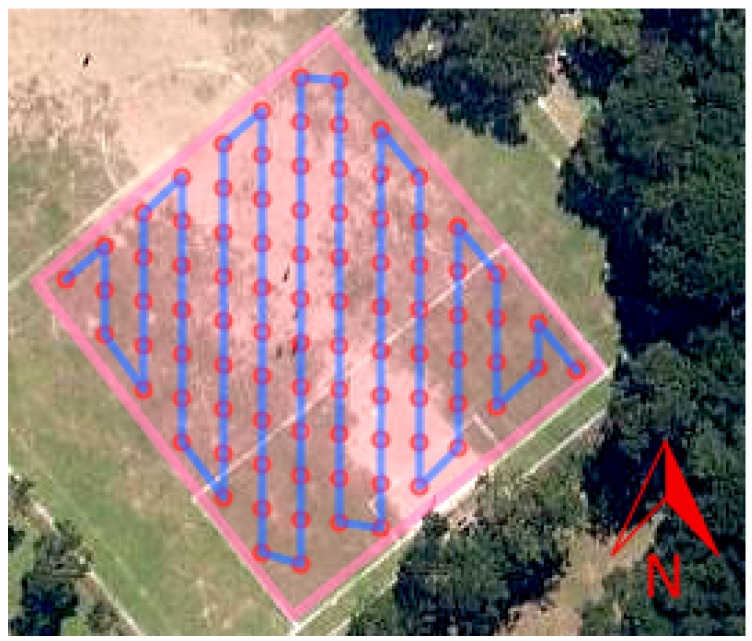
Example of generated path following magnetic north, with 5 m spacing between samples. The pink area is the region of interest, the blue lines are the expected path to be followed and the red circles are the discrete waypoints to visit.

The average euclidean norm value *T*, which is also known as the total field, is computed from 10 samples obtained for each point in order to minimize noise influence. The unit used for *T* is nanotesla. Figure 7 shows an example of the generated grid for an arbitrary mapping profile to be followed by the drone. We follow the traditional approach of calculating the euclidean norm value *T* (total field) for each point, which represents the intensity/strength of the magnetic field of a point in the 3D space, as described in [27]. Other methods may use the gradient of *T* (gradiometers), but all of these methods consider the magnitude of the signal to generate a raster of values for each pixel on the final map.

### 4.2. Plotting and Interpolation

Potential fields such as the magnetic field are continuous in space and devoid of abrupt changes and discontinuity. Therefore, an interpolator can be used to obtain a smooth continuous curve that fits on the observed points, and can fairly approximate to the real function that generated the original signal if there is sufficient known points [27]. The most used methods for interpolating geographic data are the Kriging [31], Splining [32] and Inverse Distance Weighting (IDW) interpolation [33].

By using the GPS coordinates and the mean total field *T* calculated for each position of the grid collected from the survey, we first generate a sparse 2D map, using a color map for the values of *T*.

To obtain the final map, we use a multiquadratic Radial Basis Function (mRBF) interpolator to estimate a smooth curve that passes through the scattered sample points. The choice of mRBF interpolator was made by experimenting with several different interpolation methods, including the most used ones. To evaluate the interpolators’ accuracy and also optimize their parameters, we used a two-fold cross-validation method that consisted in splitting the dataset into train and test set by randomly selecting points uniformly, using the mean squared error as metric. We concluded that that the mRBF interpolation is able to provide better results, specially when tested on noisy data. In our case, it is very important that the interpolator is able to handle noisy data due to the sensor intrinsic noise and other sources of dynamic interference that may be caused by the aerial vehicle itself or external sources.

**Figure 8:**
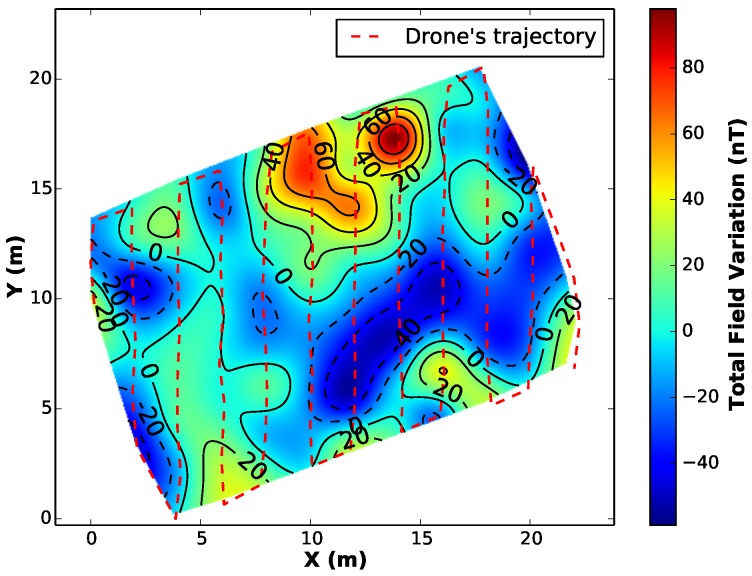
Illustration of the interpolated data represented by a coloured contour map.

Finally, we generate a regular grid of a desired resolution that can be visualized through different kind of maps, e.g., shaded reliefs, color maps or contour maps. Figure 8 depicts the resulting maps generated from a campaign.

## 5. Experimental Evaluation

Additional to the standard testing and calibration protocols, the magnetometer was subject to a few characterization procedures in order to confirm its specifications as required for this unique application.

### 5.1. Noise and Sensitivity

**Figure 9:**
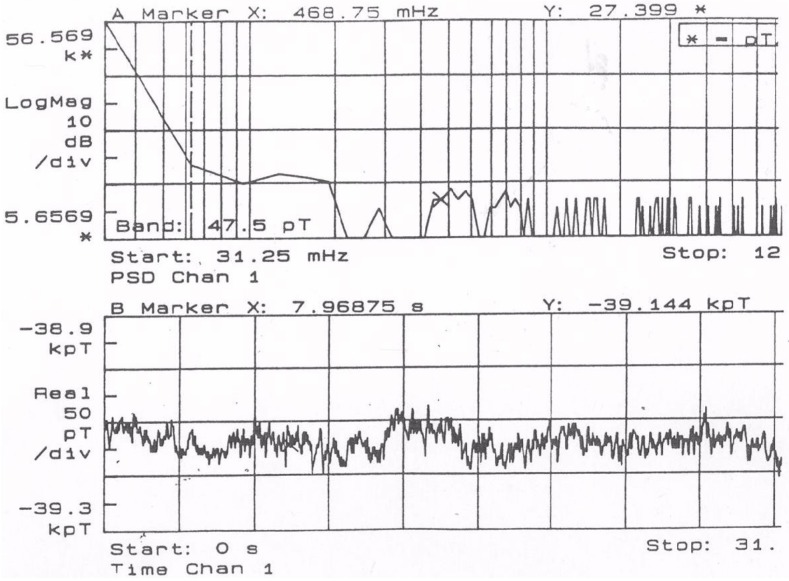
Noise level for selected CoFeSiB fluxgate sensor.

While typical commercial sensor cores present noise level of 100 pT rms in the interval of 0.1 Hz to 10 Hz, the sensor used in this experiment presents—in the same level—noise value below 47.5 pT rms (Figure 9). The sensor output (before data conversion) was measured by a digital Spectrum Analyzer (SR850, Stanford Research Systems/CA-USA). During this analysis the sensor elements were kept inside a demagnetized mu-metal shield with five layers.

For sensitivity characterization a 0.1 Hz square wave current driving a 100 cm wide triaxial Helmholtz coil was used. The current square wave was obtained from a computer controlled source calibrator (2400 SourceMeter, Keithley Tektronix/OR-USA) and a lab current amplifier with current sensors inline. A set of three 6 1/2 digit voltmeters (8846A 6.5 Digit Precision Multimeter, Fluke/WA-USA) were used to compare each channel reading. Signals as low as 0.5 nT were detected showing excellent sensitivity for the instrument.

### 5.2. Frequency Response

**Figure 10:**
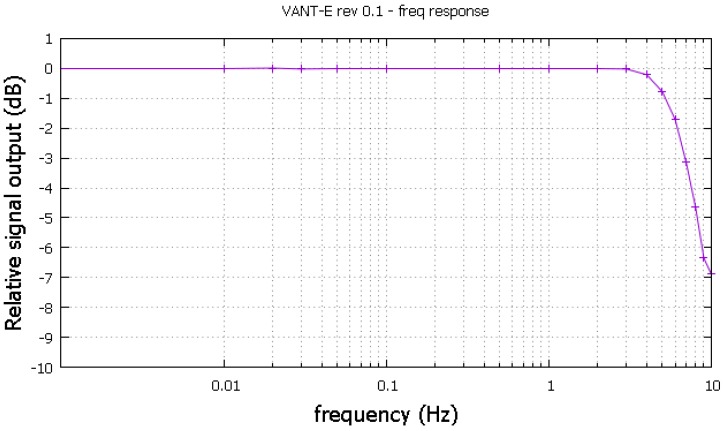
The sensor frequency response.

To test the magnetometer frequency response, a sinusoidal current sweep ranging from a few milihertz to 10 Hz was applied to the Helmholtz coil (single axis at a time). For these low frequencies direct AC rms readings are not possible due to voltmeter low band limitations. A fast DC sampling with instantaneous normalization was used instead. After some adjustments the magnetometer achieved a −3 dB cutting just above 7 Hz as shown in the Figure 10.

### 5.3. Sensor Behavior Characterization

Using a magnetometer sensor in proximity with an electrically motorized drone requires special considerations. The motor iron components and its strong operational current represent a significant source of noise, capable to disturb and even impair the intended magnetic survey. Under normal operation, the drone internal computer supplies the motor with a wide range of power levels, depending on the flight profile and attitude.

In order to empirically estimate the UAV intrinsic magnetic noise and avoid as much as possible its disturbance, a set of measures were taken around the vehicle with the help of a GSM-19T proton magnetometer. The GSM-19T is an instrument of regular use for geophysical surveys, showing great sensitivity and precision more than adequate for our noise investigation. To map all possible situations, we tested the drone at different distances from the GSM-19T exercising motor current levels ranging from off state to full power, searching for a minimum possible distance for the magnetometer to be installed where no interference could be detected. Since this is a much more sensitive base magnetometer, we found safe distances of aproximately 3 m.

We have also conducted an experiment with the developed fluxgate magnetometer. The experiment is composed by three tests, each one consisted in a mapping profile containing a single line of 6 m long aligned to the magnetic north, at a place free of sources of strong magnetic interference. Each measurement was obtained every 0.5 m along the line, where for each measurement we collected the mean norm of 10 readings from the sensor, in order to remove the high frequency noise. In all tests, the drone was positioned in the middle of the line.

The goal for this analysis was to reduce as much as possible the distance between drone and sensor, considering that a sensor hanging in a long cable can pose limitations on flight control and generate safety issues. Below a distance of one meter the disturbance was such that in some events the magnetometer was unable to provide any readings even for the lowest power levels applied. We found that distances starting from 2 m can be used depending on the drone size and motor power required for a given flight profile. Our conclusion is that beyond 3 m of separation between sensor and drone no significant change on readings was present at any power level.

In the first test, the drone was turned off and the battery cable was disconnected. The second test consisted in powering the drone, excepting the motors. Finally, in the third test, the motors were rotating at moderate speed, without taking off the ground.

By analyzing the results, we observed a peak at approximately the 1.0 m mark. So we safely assume the distance of the next stable measurement, as a minimum distance to locate the sensor. Finally we decided to add 10 cm extra for cautiousness, ending up with the sensor being located at 1.1 m of the drone. Based on this simple (and conservative) approach, our observations have shown that the impact of the drone’s airframe, electronics and power levels were negligible at the chosen distance and position. Others works in the literature, for example [34], report on the use of booms of similar size (considering the distance to the motors).

This distance is very important because the magnetic field of the robot will change depending on payload and thrust applied to motors, so incorrectly setting this distance will interfere directly with the measurements in a non-predictable way. We have not conducted an exhaustive characterization of the setup, as at this moment the focus was to find a compromise solution that would guarantee the system operation, allowing us to demonstrate the feasibility of the concept. Nevertheless, measuring the sensitivity of the complete setup is part of a future work.

**Figure 11:**
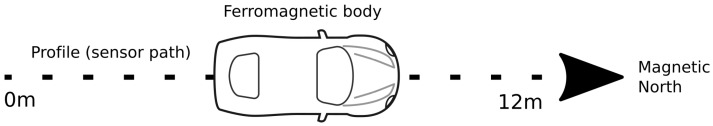
Profile used for testing a basic survey with the drone magnetometer.

**Figure 12:**
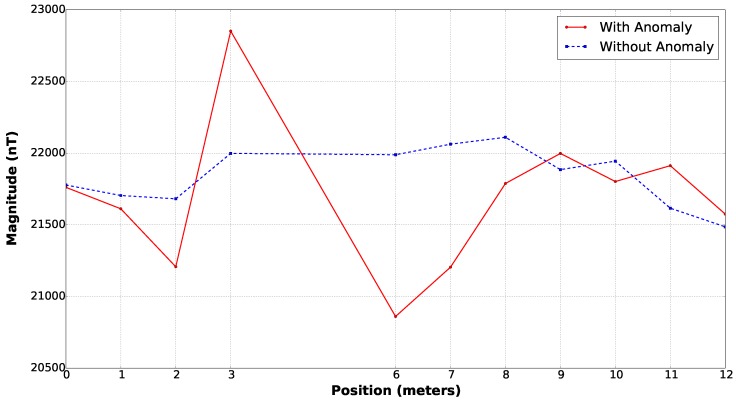
Survey along a test profile aligned to the magnetic north. Two survey has been carried, with and without a ferromagnetic test body containing approximately 300 kg of low carbon.

Finally, the last evaluation applied was a small survey over a controlled site submitting the sensor to real environment conditions of local noise and gradients. A 12 m profile where a ferromagnetic container of half cubic meters and 300 kg was placed over the magnetometer path as depicted on Figure 11. The results for this survey (Figure 12) exhibit an anomaly having an amplitude of approximately 2000 nT next to the position the container was placed.

### 5.4. UAV Sensor Mounting

Small aerial robots have serious payload limitations, and their small size also creates a challenge to accommodate a large number of sensors and payload. The developed magnetometer has a weight of 89 g (head) and 174 g (PCB with case). Due the light weight nature of this sensor, typical pendulum carrying wasn’t possible: small wind perturbations greatly alter the sensor position and angle, thus decreasing sensor accuracy.

The way the sensor is mounted on the drone is key to having correct measurements. We tested different mountings such as pendulum and vertical mountings. Despite the pendulum mounting don’t had any serious impact in aircraft mobility, wind and even the normal movement of the drone generated a unpredictable motions on the sensor, thus generating extra noise. A vertical mounting do not prove to be robust enough on this type of drone due the expected distance between the sensor and the drone body.

**Figure 13:**
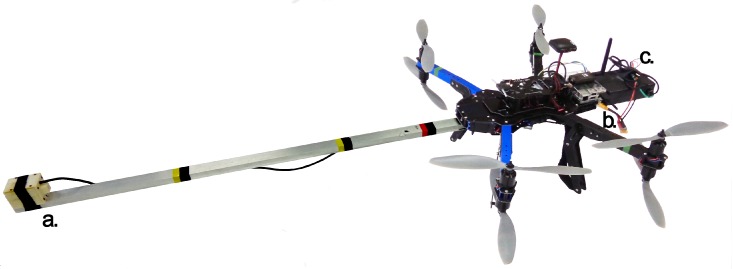
Close up of chosen Unmanned Aerial Vehicle (UAV): X8+ from 3DR. Important components are highlighted on the picture. (**a**) Sensor head with an 1.1 m aluminium boom; (**b**) compainion computer: Raspberry Pi 2; (**c**) sensor body.

Given those limitations we proposed a “boom” mounting. A 1.1 m lightweight 100% aluminum bar pointing at the front of the robot was the more stable mounting. The magnetic permeability of aluminum is known to be close to $\mu_{0}$, making it a good material for the magnetometer mounting. This mounting can be seen on Figure 13 on a X8+ drone from 3DR. Also a companion computer and extra batteries needed to be added to acquire and process the sensor data accurately. A Raspberry Pi 2 computer was connected to the *Pixhawk* control board to process sensor information when a survey point was reached.

All the weight on the aircraft needed to be balanced before flight to avoid misbehavior and control problems during flight. This balancing was done on the *X* and *Y* axes of the aircraft, carefully moving all components of the system until the drone stays still on his center of gravity.

### 5.5. UAV Field Experiment

In order to validate our technique, we performed a complete survey using the proposed methodology. All the steps required for the mapping and data processing phases were performed automatically by the robot and processing software with little to no human interaction.

**Figure 14:**
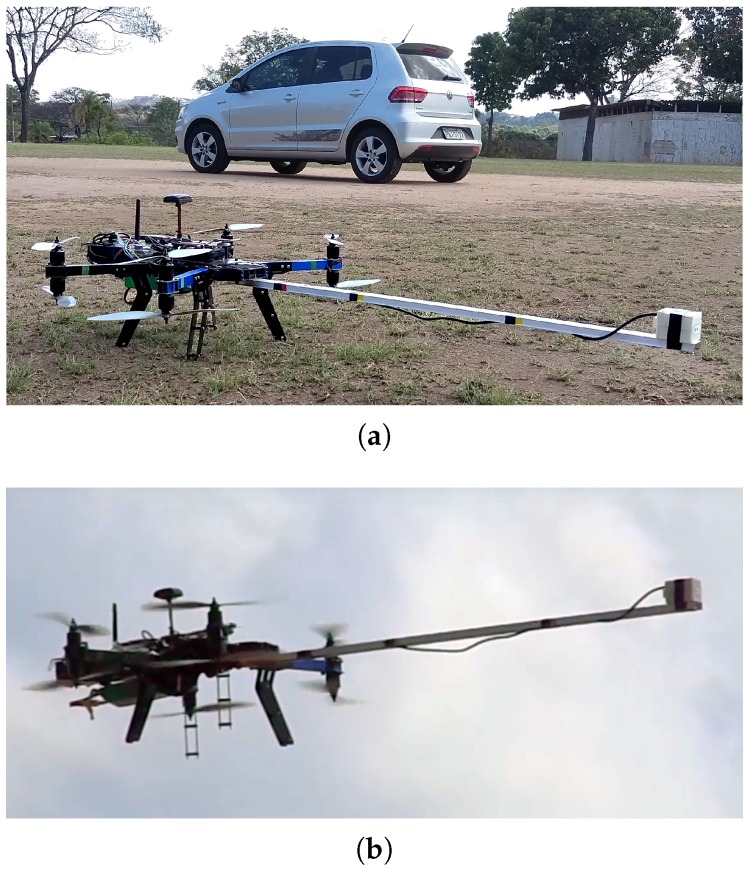
Field experiment scene. (**a**) The sensor components are being carried by the octarotor, and its core is supported by a pole of 1.1 m, in order to avoid as much as possible the magnetic interferences caused by the drone itself. In the background, the car used as the magnetic anomaly originator; (**b**) UAV flying a mission.

The objective of this experiment was to generate consistent maps, where one is able to identify an artificial anomaly caused by a metallic structure placed at a known position. A 2.10 m × 3.00 m car was used as an anomaly. The chosen location for the survey was the middle of a soccer field, far away from other sources of magnetic interference such as power lines or big metal structures. Figure 14 depicts the experiment setup. A video illustrating the complete execution of a survey campaign is available at [35].

**Figure 15:**
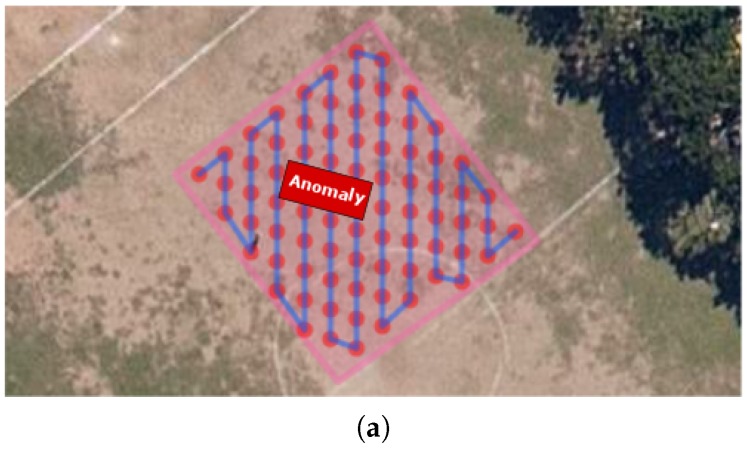

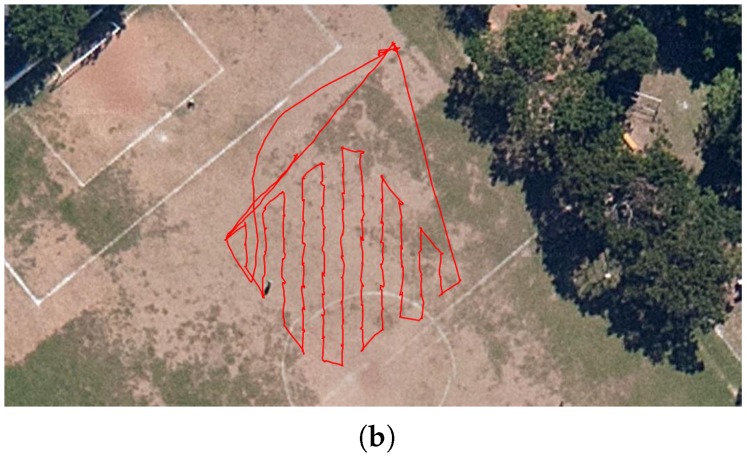
Field experiment survey path. (**a**) The planned path with a 2 m separation between survey points on an area of 381 m^2^. The red dots are the survey points where the robot hovers ($t_{h}=7$ s), and the red rectangle the position of the artificial anomaly; (**b**) The real executed trajectory of the robot on red.

The mapping profile created for this experiment has a 2 m of separation between survey points, and 7 s hover time over an area of 381 m^2^. The survey length was 181 m. At every survey point the robot faced the sensor to the magnetic north. The planned survey path and the executed one can be seen on Figure 15.

**Figure 16:**
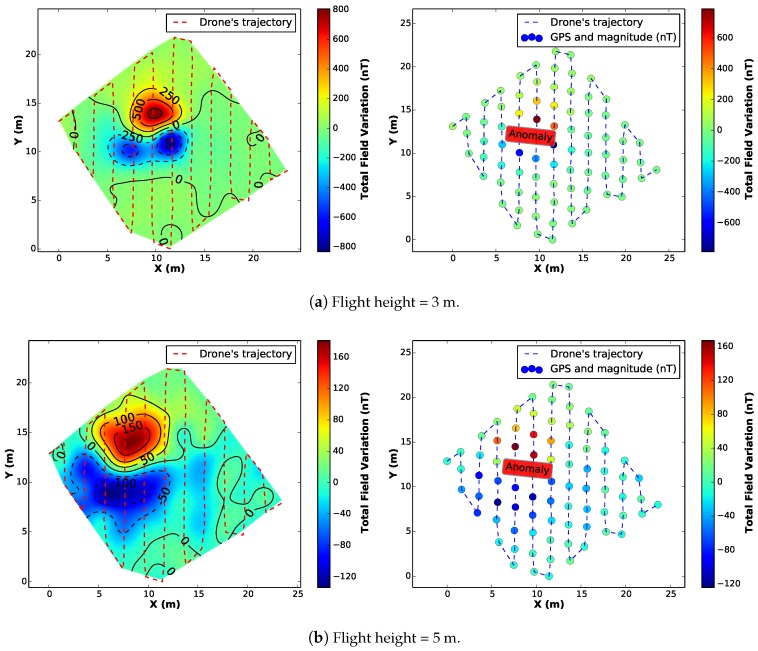

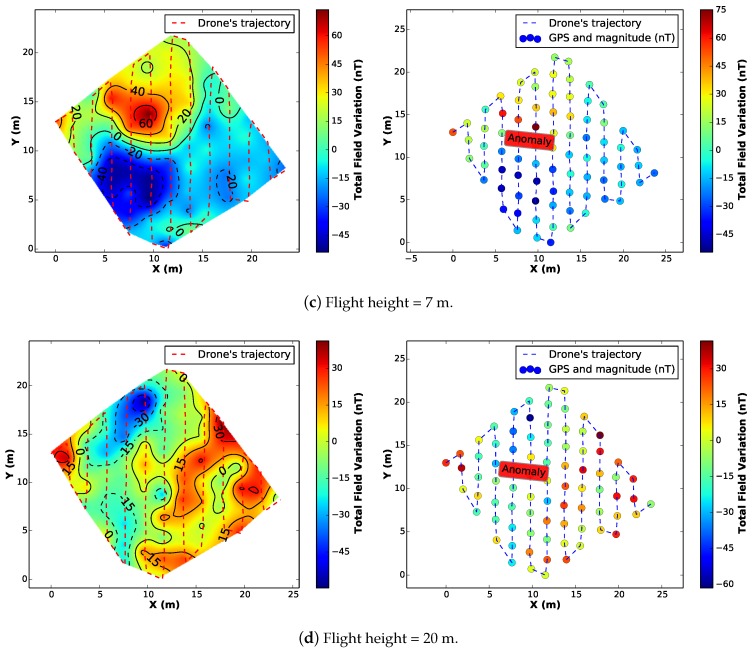
Generated maps from the complete survey at different heights with a artificial anomaly placed in the hightlighted position on the right (sparse) map.

We executed the same profile configuration for 4 different surveys at 3, 5, 7 and 20 m of height above the ground. Figure 16 presents the results of the aeromagnetic maps.

By comparing the interpolated maps from all the surveys, it is clear that the magnetometer was able to detect the variation of the magnetic field in the vehicle’s surroundings, leaving an expected dipolar magnetic signature on the generated map, specially for the first case (flight height = 3 m). However, as expected, with a greater distance between the sensor and the anomaly (flight height = 20 m), the map looses quality/resolution and the anomaly is undetectable.

## 6. Conclusions and Future Work

### 6.1. Conclusions

The interest and research in UAVs has been increasingly growing, specially due to the decrease in cost, weight, size and performance of actuators, sensors and processors. These type of vehicles clearly have their niche of applications, being one of their main advantages a privileged view from above. Considering the mining industry, it is possible to use such vehicles in the prospection of minerals of commercial interest beneath the ground.

In this context, we discussed the development of a state-of-the-art three-axis fluxgate magnetometer to be used in autonomous aeromagnetic surveys. The electronic was built aiming to reduce its size and power consumption, and the sensor housing was assembled far from the electronics in order to allow enough distance from important sources of magnetic noise, such as the vehicle’s motors. Due to the sensor small size and weight, it is very sensitive to noise generated from fast rotational movements and vibration. We proposed the use of rotary-wing autonomous aerial robots that can hover over survey points. This limitation makes it impractical for mounting this sensor on other types of aircraft such as fixed-wing aircrafts.

For the particular task of aeromagnetic surveys, we have proposed the use of modified version of the Maza et al. [22] path planning algorithm, which is based on the lawnmower coverage pattern. It was modified to subdivide the path on several small size segments and to customize survey angle to match the magnetic north or another arbitrary angle. Also we proposed a method to overcome GPS accuracy on surveys, changing the visiting sequence of those survey points.

Through an extensive set of experiments performed to determine the mapping profile parameters, map generation methodology and adequate vehicle’s mounting configuration, we were able to successfully generate meaningful magnetic maps. Our method is highly automated, and the system is easily reproducible and scalable to multiple drones with small addition in costs.

### 6.2. Future Work

The work has several future directions that can be pursued in order to improve the methodology, which includes the extension of the proposed technique to multi-robot ensembles and to environments with obstacles, both providing better representations of real-world scenarios.

Furthermore, every survey campaign has unique characteristics, in this sense, on future work we aim to improve the parameter selection for surveys such as hover time, sample size and distance between survey points. We also intend to evaluate how the influence of different sensor mounting positions affect accuracy of data acquisition.

Future work will also involve the application of the proposed methodology on a prospective mining scenario, comparing results to known previous generated maps with conventional methods.

## Figures and Tables

**Table 1:** Developed magnetometer specifications summary.

Full Scale Range	± 60 μT
Resolution	<0.5 nT
Frequency Response	DC to 4 Hz (−1 dB)
Overall Linearity	0.02%
Precision	Better than 10 nT
Power consumption	≈0.9 W @ 12 VDC
Weight	≈89 g (head) + 174 g (body)
Size Head	5.5 cm × 4 cm × 4 cm
